# Supplementary material for: Carbon Dioxide Sensing—Biomedical Applications to Human Subjects
Source: Sensors (Basel). 2021 Dec 28;22(1):188. doi: 10.3390/s22010188 (PMC8749784; doi:10.3390/s22010188)
Supplement: Supplementary file 1 [file sensors-22-00188-s001.zip › sensors-1476836 Supplementary.pdf]

Supplementary Material (S1)
The Dynamic of Transcutaneous Sensing: A Closed Chamber Model

Abstract

The aim of this document is to give the complete details and related calculations of a simplified transcutaneous carbon dioxide (CO_2) sensor model using a closed chamber approach. The key results of this short study are presented in Section 4.2.3 of the main article.

1 The Model

The simplified model of the transcutaneous carbon dioxide (CO_2) sensor under study is presented in Figure S1.

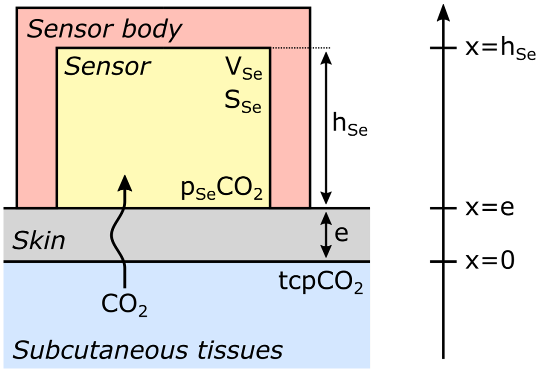

Figure S1: Sensor model.

In this model, the subcutaneous tissues are considered as a homogeneous, semi-infinite medium occupying the $x \leq 0$ space volume. The subcutaneous tissues can be thought of as a CO_2 reservoir with a constant partial CO_2 pressure equal to tcpCO_2. Between $x = 0$ and $x = e$ is the skin membrane, of thickness e and diffusivity D ($m^2.s^{-1}$) towards CO_2. In this skin model, the sub-cutaneous tissues and skin barrier are only used as a representation, and the skin barrier does not represent literally the stratum corneum. Consequently, the e and D

parameters can hardly be measured experimentally since they are mere model parameters.

On top of these two layers is positioned a sensor consisting in an inner sensing medium of volume V_{Se}, surface in contact with the skin S_{Se} and height $h_{Se} = V_{Se}/S_{Se}$. This sensor is in turn surrounded by a gas-tight enclosure, preventing gaseous exchanges between the sensing medium and the outside—except for those with the skin surface, of course.

2 Inner Sensing Medium

In the following calculations, the inner sensing medium is considered as an homogenous air volume. While this assumption is more than justified in the case of Non Dispersive Infra-Red (NDIR) or (photo-)acoustic sensors, it seems more debatable in the case of conductometric or dye-based sensors. Yet, every sensing medium can be viewed as a potential CO_2 reservoir through Henry's law, and thus be considered as an air volume.

For instance, if the sensing medium is water—*e.g.* in the case of a wet conductometric sensor—with a water volume V_{water} of height h_{water}, this latter water volume is equivalent to an air volume $V_{air,eq}$ of height $h_{air,eq}$ such that the amount of CO_2 dissolved in the water volume $n_{CO_2,water}$ (mol) is the same as that in the equivalent air volume $n_{CO_2,air,eq}$ for a given pCO_2:

$$n_{CO_2,air,eq}(pCO_2) = n_{CO_2,Water}(pCO_2) \tag{1}$$

with

$$\begin{aligned} & n_{CO_2,air,eq} = \frac{pCO_2 \cdot V_{air,eq}}{R \cdot T} \quad \text{(ideal gas law)} \\ & \mathcal{C}_{CO_2,water} = K_0 \cdot pCO_2 \quad \text{(Henry's law)} \\ & n_{CO_2,Water} \triangleq V_{water} \cdot \mathcal{C}_{CO_2,water} \\ & V_{Water} = S_{Se} \cdot h_{water} \quad \text{and} \quad V_{air,eq} = S_{Se} \cdot h_{air,eq} \end{aligned} \tag{2}$$

wherein R is the ideal gas constant (J.K^{-1}.mol^{-1}), T the temperature (K) and K_0 (mol.m^{-3}.Pa^{-1}) is the solubility of CO_2 in water as reported by Weiss[**weiss1974**]. Combining the above equations yields:

$$h_{air,eq} = h_{water} \cdot R \cdot T \cdot K_0 \tag{3}$$

Taking Weiss' value for K_0 in pure water at 293 K under 1 atm yields:

$$h_{air,eq} \approx 0.952 \cdot h_W \tag{4}$$

and a similar train of thought can be applied to other media. Using Henry's law, it is thus always possible to convert from a given sensing medium into an equivalent air volume as is done above for water.

Then, it should be emphasised that only the equivalent height h_{Se} of the sensor really matters. Indeed, no matter what the actual inner volume of the sensor looks like, only its volume to surface ratio matters, as is detailed below.

Finally, one should bear in mind that the CO_2 diffusion process into the sensing medium is also neglected. Indeed, it is assumed in the remainder of this article that as soon as CO_2 crosses the skin barrier and reaches the sensor itself, it readily diffuses into it. Thus the pCO_2 inside the sensing medium is considered to be homogenous and only a function of the amount of CO_2 diffusing through the skin. This assumption is more than justified in first approximation when considering the response times of the different sensing techniques presented in Section 2 of the review article. Indeed, most of them easily reach response times below 1 min, which is far below the response times of the simple model presented here for thicknesses h_{Se} above 100 µm, as is presented below—see Section 4.

3 Diffusion Through the Skin

The CO_2 diffusion across the skin barrier can be studied using Fick's first law of diffusion:

$$J_S = -D \cdot \frac{d\mathcal{C}_{CO_2}(x)}{dx} \tag{5}$$

wherein J_S is the CO_2 diffusion flux per unit area (mol.m^{-2}.s^{-1}), D the diffusivity of skin toward CO_2 (m^2.s^{-1}), and $\mathcal{C}_{CO_2}$ the CO_2 concentration (mol.m^{-3}) inside the membrane. With the hypothesis ($\mathcal{H}_1$) that there is no CO_2 accumulation inside the skin itself—*i.e.* that the capacitive effect of the skin is negligible compared to that of the sensing medium—J_S can be considered constant along the x axis, leading to:

$$J_S = \frac{D}{e} \cdot (\mathcal{C}_{CO_2}(x=0) - \mathcal{C}_{CO_2}(x=e)) \tag{6}$$

Since $x = 0$ corresponds to the subcutaneous tissues wherein pCO_2=$tcpCO_2$ and $x = e$ corresponds to the inner sensing medium wherein pCO_2=$p_{Se}CO_2$:

$$\begin{cases} \mathcal{C}_{CO_2}(x=0) = K_{skin,CO_2} \cdot tcpCO_2 \\ \mathcal{C}_{CO_2}(x=e) = K_{skin,CO_2} \cdot p_{Se}CO_2 \end{cases} \tag{7}$$

with K_{skin,CO_2} the solubility of CO_2 into the skin (mol.m^{-3}.Pa^{-1}). Besides, under ($\mathcal{H}_1$):

$$\frac{dn_{CO_2,Se}}{dt} = S_{Se} \cdot J_S \tag{8}$$

wherein $n_{CO_2,Se}$ is the amount of CO_2 in the inner sensing medium (mol), given by:

$$n_{CO_2,Se} = \frac{p_{Se}CO_2 \cdot V_{Se}}{R \cdot T} \quad \text{(ideal gas law)} \tag{9}$$

Equations 6–9 yields:

$$\frac{dp_{Se}CO_2}{dt} = \frac{D}{e \cdot h_{Se}} \cdot R \cdot T \cdot K_{skin,CO_2} \cdot (tcpCO_2 - p_{Se}CO_2) \tag{10}$$

Additionally, K_{skin,CO_2} can be estimated from the solubility of CO_2 in various biological tissues[**wright1934**, **nichols1957**, **scheuplein1976**, **gill1988**] leading to a $R \cdot T \cdot K_{skin,CO_2}$ factor in the 0.6–1.0 range. Close to these values, Scheuplein *et al.*[**scheuplein1976**] present a $R \cdot T \cdot K_{skin,CO_2}$ near 1.5 for the stratum corneum. Again though, the skin membrane of our model does not necessarily represent the stratum corneum alone, but is only a model for the different skin layers that may hinder CO_2 diffusion. Consequently, we chose to take the $R \cdot T \cdot K_{skin,CO_2}$ product equal to 1 in the following developments. Another possibility would be to simply integrate it inside the e/D ratio, leading to identical conclusions in the end, at the cost of an additional complexity in the writing. Thus, we can reasonably discard it, leading to the following simplified differential equation:

$$\frac{dp_{Se}CO_2}{dt} = \frac{D}{e \cdot h_{Se}} \cdot (tcpCO_2 - p_{Se}CO_2) \tag{11}$$

which—considering the initial condition $p_{Se}CO_2(t=0) = 0$—has a unique solution:

$$p_{Se}CO_2(t) = tcpCO_2 \cdot \left(1 - exp\left(-\frac{t}{\tau}\right)\right), \quad \text{with } \tau = h_{Se} \cdot \frac{e}{D} \tag{12}$$

This formulation is essentially the same as that found by Chatterjee *et al.*[**chatterjee2015**], although they used a more sophisticated model taking into account the CO_2 production inside the skin, leading to an additional β/α factor in Equation (6) of their work.

4 Response Time

As mentioned in Section 1, the e and D parameters cannot be measured experimentally since they are only model parameters. However, the e/D fraction can be estimated from the CO_2 exhalation rate through the skin—Q_S ($m^3.m^{-2}.s^{-1}$)—defined as:

$$Q_S = \frac{1}{S_{Se}} \cdot \frac{dV_{CO_2,Se}}{dt} \tag{13}$$

wherein $V_{CO_2,Se}$ is the volume that would occupy the CO_2 contained inside the sensing medium if considered as pure CO_2 under total ambient pressure p_0, given by:

$$\frac{V_{CO_2,Se}}{V_{Se}} = \frac{p_{Se}CO_2}{p_0} \tag{14}$$

yielding:

$$Q_S = \frac{h_{Se}}{p_0} \cdot \frac{dp_{Se}CO_2}{dt} \tag{15}$$

Taking the first derivative of $p_{Se}CO_2$—see Equation 12—with respect to time, and evaluating it at $t = 0$ leads to:

$$\tau = \frac{h_{Se}}{Q_S} \cdot \frac{tcpCO_2}{p_0} \quad \text{or} \quad \frac{e}{D} = \frac{tcpCO_2}{Q_S \cdot p_0} \tag{16}$$

Several works on the determination of Q_S for CO_2 across human skin can be found in the literature—see Table 2 in the review article—and a value of 100 $cm^3.m^{-2}.h^{-1}$ can reasonably be assumed. Since most subjects in the aforementioned works were healthy, we can also assume a normal capnia of 5.3 kPa (or 40 mmHg)[**nunns**]. In these conditions, and $p_0 = 1$ atm:

$$\frac{e}{D} \approx 1.90 \times 10^6 \text{ s.m}^{-1} \tag{17}$$

In other words, if the sensor has a height of 1 mm, τ will be near 1900 s, or about 32 min, and a 95% response will be achieved after 1 h 35 min—or $3 \cdot \tau$. These calculations are summarised in Figure S2.

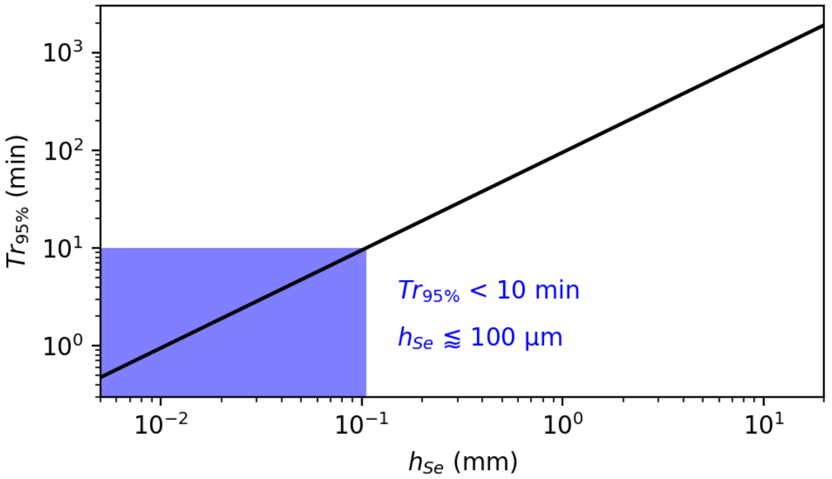

Figure S2: 95% response time ($Tr_{95\%} \approx 3 \cdot \tau$) for a sensor of height h_{Se}. The blue area underlines the portion of the line with a response time below 10 min, which corresponds to a sensor height below approximately 100 µm.

Note: Q_S is of course time-dependent, especially during transcutaneous monitoring with the presented closed-chamber sensor model—as it will decrease with a diminishing $tcpCO_2$ / $p_{Se}CO_2$ gradient. However, since only its value at $t = 0$ has been used in the end, and since it is also the only one reported in the literature, the $Q_S(t)$ notation has been dropped.
